# MiR-34a-5p promotes the multi-drug resistance of osteosarcoma by targeting the CD117 gene

**DOI:** 10.18632/oncotarget.8546

**Published:** 2016-04-01

**Authors:** Youguang Pu, Fangfang Zhao, Haiyan Wang, Wenjing Cai, Jin Gao, Yinpeng Li, Shanbao Cai

**Affiliations:** ^1^ Cancer Epigenetics Program, Anhui Cancer Hospital, West District of Anhui Provincial Hospital, Hefei 230031, Anhui, China; ^2^ Department of Clinical Geriatrics, Anhui Provincial Hospital, Hefei 230031, Anhui, China; ^3^ Indiana University School of Medicine, Indianapolis, IN 46202, USA; ^4^ Department of Radiation Oncology, Anhui Cancer Hospital, West District of Anhui Provincial Hospital, Hefei 230031, Anhui, China; ^5^ Xinxiang Medical University, Xinxiang 453000, Henan, China; ^6^ Department of Orthopedic Surgery, Anhui Cancer Hospital, West District of Anhui Provincial Hospital, Hefei 230031, Anhui, China

**Keywords:** miR-34a-5p, CD117, multi-drug resistance, osteosarcoma

## Abstract

An association has been reported between miR-34a-5p and several types of cancer. Specifically, in this study, using systematic observations of multi-drug sensitive (G-292 and MG63.2) and resistant (SJSA-1 and MNNG/HOS) osteosarcoma (OS) cell lines, we showed that miR-34a-5p promotes the multi-drug resistance of OS through the receptor tyrosine kinase CD117, a direct target of miR-34a-5p. Consistently, the siRNA-mediated repression of CD117 in G-292 and MG63.2 cells led to a similar phenotype that exhibited all of the miR-34a-5p mimic-triggered changes. In addition, the activity of the MEF2 signaling pathway was drastically altered by the forced changes in the miR-34a-5p or CD117 level in OS cells. Furthermore, si-CD117 suppressed the enhanced colony and sphere formation, which is in agreement with the characteristics of a cancer stem marker. Taken together, our data established CD117 as a direct target of miR-34-5p and demonstrated that this regulation interferes with several CD117-mediated effects on OS cells. In addition to providing new mechanistic insights, our results will provide an approach for diagnosing and chemotherapeutically treating OS.

## INTRODUCTION

MiRNAs are a class of small non-coding regulatory RNA molecules that have been shown to be involved in a wide range of biological processes [1]. The dysregulation of miRNAs has been associated with the development of numerous diseases, including cancer. The abnormal expression of miRNAs in cancer contributes to every aspect of tumor biology [2, 3], including drug resistance [4], which remains a major obstacle to effective treatment. Numerous mechanisms have been found that mediate drug resistance, including decreased intracellular drug accumulation, drug inactivation, enhanced DNA repair, perturbations in signal transduction pathways, apoptosis and autophagy related drug resistance, miRNA dysregulation and cancer stem cell (CSC)-mediated drug resistance [5]. To date, much effort has been exerted to analyze the role of miRNAs in the development of drug resistance in various types of cancers. For instance, a correlation between the overexpression of miR-21 in colorectal cancer and decreased sensitivity to fluorouracil (5-FU) was reported [6]. In addition, miR-130a was up-regulated in SKOV3/DDP cells compared with SKOV3 cells, and miR-130a inhibition could overcome cisplatin resistance by regulating the MDR1/P-gp pathway [7]. The expression of miR-140 was involved in the drug resistance to osteosarcoma (OS) xenografts through reduced cell proliferation *via* G1 and G2 phase arrest [8].

An association has previously been reported between miR-34a, a well-studied miRNA, and several types of cancer, including Ewing's sarcoma [9] and colorectal cancer [10], among others. In addition, miR-34a-5p has been reported to be a direct transcriptional target of p53 and is down-regulated in several tumors [11, 12]. Moreover, miR-34a-5p has been found to inhibit cell invasion and migration *in vitro* [13–16], which suggested that miR-34a-5p might play a role in inhibiting tumor recurrence.

OS is the most common malignant primary bone tumor in children and adolescents [17, 18], but the mechanism underlying OS drug resistance remains unknown. In addition, despite the above extensive studies on miR-34a-5p, the relationship between miR-34a-5p and OS drug resistance is still unclear. In this study, we found that miR-34a-5p promotes multi-drug resistance in OS cells using a systematic analysis to compare multi-drug sensitive (G-292 and MG63.2) OS cell lines to resistant (SJSA-1 and MNNG/HOS) OS cell lines. We further showed that miR-34a-5p promotes OS multi-drug resistance *via* repression of the CD117 gene, a newly identified direct target of miR-34a-5p. The CD117 gene encodes a receptor tyrosine kinase (RTK) belonging to the transmembrane RTK family [19] that is involved in the tumorigenesis of several neoplasms. CD117 can be expressed in a wide variety of malignant tumors, such as chronic myeloid leukemia, gastrointestinal stromal tumor, malignant melanoma, seminoma, and adenoid cystic carcinoma of the salivary gland [20].

In this study, we also determined that the MEF2 signaling pathway is affected by miR-34a-5p *via* repression of the CD117 gene. The MEF2 signaling pathway has roles in different tissues through effects on cell differentiation, proliferation, apoptosis, migration, shape and metabolism. Altered MEF2 activity plays a role in human diseases and has been implicated in the development of several cancer types [21]. Taken together, our findings will provide a theoretical guide for a clinical therapy to combat OS drug resistance as well as provide new mechanistic insights into OS drug resistance.

## RESULTS

### MiR-34a-5p promotes multi-drug resistance in OS cells

**Figure 1:**
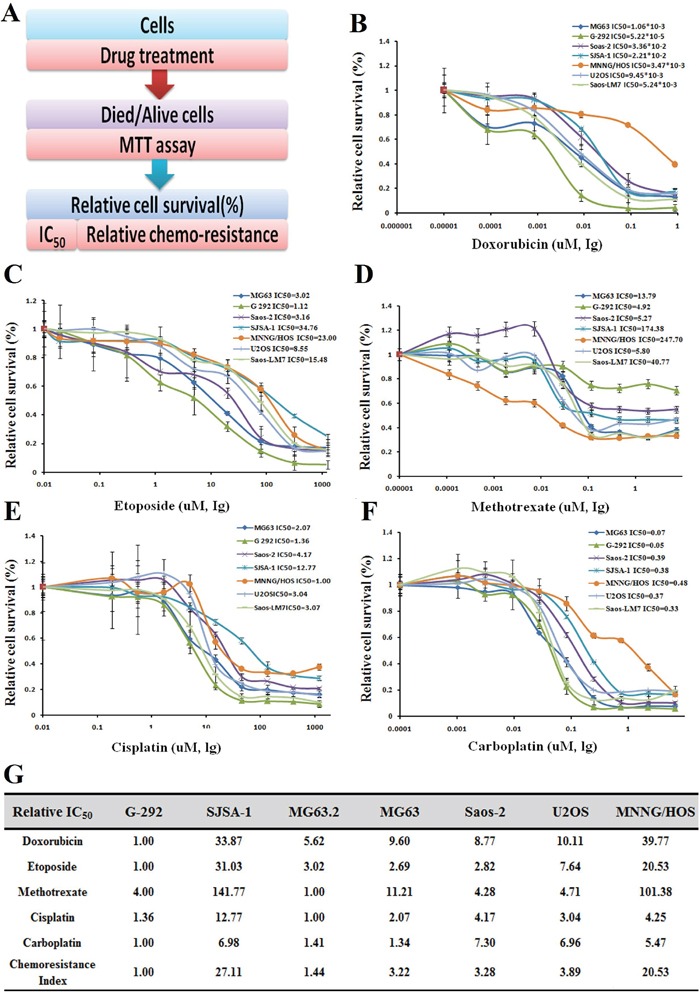
Drug resistance profiling of seven osteosarcoma cell lines **A.** Experimental scheme. **B-F.** IC_50_ values of the five indicated chemotherapeutics for seven osteosarcoma cell lines. The cell survival rates were calculated as percentages relative to the mock treatment and plotted against lg μM of drug. **G.** The IC_50_ (−fold) values relative to those of the most sensitive cell cine (G-292) are presented in the table.

**Figure 2:**
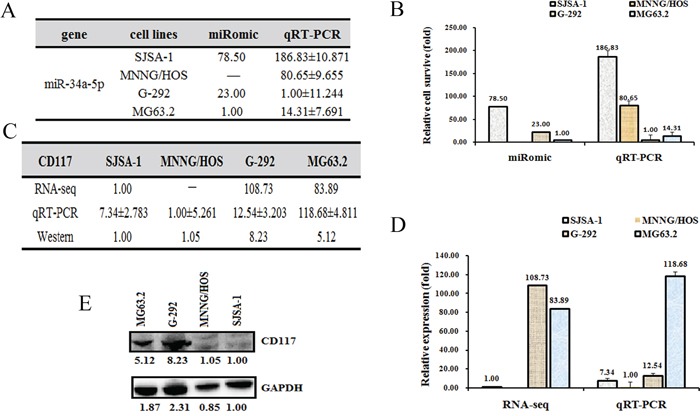
The CD117 level is higher in G-292 and MG63 2 cells than in SJSA-1 and MNNG/HOS cells. The relative miR-34a-5p level (fold) in G-292 and MG63.2 cells versus SJSA-1 and MNNG/HOS cells measured by both miR-omic and qRT-PCR analyses is shown in a table **A.** and those measured by qRT-PCR are shown in a plot **B.** The relative level (fold) of the CD117 gene in G-292 and MG63.2 cells versus SJSA-1 and MNNG/HOS cells are summarized in a table **C.**, with a plot showing the miR-omic and qRT-PCR analyses **D.** and a figure showing the western blot analysis **E.** “-” indicates no detection in the omic analysis.

As follows, the drug resistance of seven OS cell lines (G-292, SJSA-1, MG63.2, MG63, Saos-2, U2OS, and MNNG/HOS) to doxorubicin (Dox), etoposide (Etop), methotrexate (MTX), cisplatin (CDDP), and carboplatin (Carb) was evaluated by IC_50_ profiling, these drugs are frequently used for OS therapy. As indicated by the drug resistance index, G-292 and MG63.2 were the most multi-drug sensitive cell lines, with the lowest IC_50_ values found against Dox, Etop and Carb for G-292 and against MTX and CDDP for MG63.2; the relative drug resistance indexes of G-292 and MG63.2 were 1.00 and 1.44, respectively. In contrast, SJSA-1 and MNNG/HOS were the most resistant cell lines with relative drug resistance indexes of 27.11 and 20.53, respectively (Figure 1). From a RNA-seq-based miR-omic analysis of the G-292, MG63.2 and SJSA-1 cell lines, we found that more than twenty miRNAs were differentially expressed by more than two-fold. Among them, miR-34a-5p was one of the most differentially expressed miRNAs in these cells. The expression of miR-34a-5p was relatively higher in SJSA-1 and MNNG/HOS cells than in G-292 and MG63.2 cells (Figure 2A and 2B). The results suggested that miR-34a-5p might promote the multi-drug resistance of OS cells.

### CD117 is a direct target of miR-34a-5p in OS cells

To identify the target genes of miR-34a-5p that are related to the multi-drug resistance of OS cells, we predicted the target genes of miR-34a-5p using the following websites: Targetscan, miRDB and microRNA.org. Several common genes were found, and the expression of these genes was then measured at both the mRNA and protein levels. The CD117 gene was selected for further study mainly because its expression is correlated with multi-drug resistance, i.e., the CD117 level was higher in G-292 and MG63.2 cells than in SJSA-1 and MNNG/HOS cells at both the mRNA and protein levels (Figure 2C, 2D and 2E).

**Figure 3:**
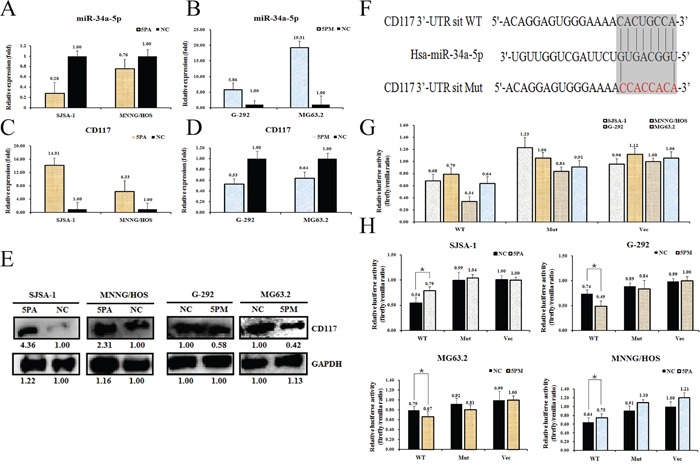
CD117 is a direct target of miR-34a-5p in osteosarcoma cells Level of miR-34a-5p **A** and **B.** CD117 mRNA **C** and **D.** and protein **E.** levels in the miR-34a-5p mimic (5PM)-transfected G-292 and MG63.2 cells and the miR-34a-5p antagomiR (5PA)-transfected SJSA-1 and MNNG/HOS cells versus the negative control (NC) cells, as determined by qRT-PCR or western blot analyses. Sequences in the UTR region of the CD117 gene targeted by miR-34a-5p, with the hatched section showing the combined area **F.** The relative luciferase activities (fold) of the reporter with the wild-type (WT) or mutant-type (Mut) CD117-UTR or without the UTR (Vec) were determined in the osteosarcoma cells transfected with the miR-34a-5p mimic (in G-292 and MG63.2), antagomiR (in SJSA-1 and MNNG/HOS) or Mock **G** and **H.** sequences. The Renilla luciferase activity of a co-transfected control plasmid was used as a control for the transfection efficiency. The representative results from three independent experiments are shown. *P value<0.05 by Student's *t*-test.

We then determined the CD117 level in miR-34a-5p mimic-transfected G-292 and MG63.2 cells and antagomiR-transfected SJSA-1 and MNNG/HOS cells for comparison with the level in the NC (scramble sequence control)-transfected cells. As shown in Figure 3A and 3B, the transfection of an miR-34a-5p mimic in G-292 and MG63.2 cells increased the miR-34a-5p level to approximately 5.86- and 19.31-fold, respectively, whereas the transfection of an miR-34a-5p antagomiR in SJSA-1 and MNNG/HOS cells significantly decreased the miR-34a-5p level to 28% and 76%, respectively. Consistent with the changes of the miR-34a-5p level, the miR-34a-5p antagomiR transfection increased both the protein and mRNA levels of CD117 in SJSA-1 and MNNG/HOS cells (Figure 3C, 3D and 3E). As expected, transfection of the miR-34a-5p mimic down-regulated the mRNA and protein levels of CD117 (Figure 3C, 3D and 3E) compared with those in the NC-transfected G-292 and MG63.2 cells.

To further confirm that CD117 is a direct target of miR-34a-5p, we cloned the fragment corresponding to nucleotides 760-1311 of the wild-type or mutant 3′-untranslated region (UTR) of the CD117 gene at the downstream of the *Renilla* luciferase gene in the pGL3-control vector (Promega) to create pGL3-CD117 UTR WT or pGL3-CD117 UTR Mut, respectively (Figure 3F). These constructs were transfected into G-292, MG63.2, SJSA-1 and MNNG/HOS cells to determine whether CD117 is a direct target of miR-34a-5p. We found that pGL3-CD117-UTR WT led to significantly higher luciferase activity in SJSA-1 and MNNG/HOS cells than in G-292 and MG63.2 cells (Figure 3G). Furthermore, the luciferase activity of pGL3-CD117-UTR WT, but not that of the other two constructs, was induced in the antagomiR-transfected SJSA-1 and MNNG/HOS cells and inhibited in the mimic-transfected G-292 and MG63.2 cells (Figure 3H). Taken together, these results led us to conclude that CD117 is indeed a direct target of miR-34a-5p and may play roles in OS drug resistance. These data are consistent with Siemens et al.'s report [22].

### CD117 expression negatively correlates with drug resistance in OS cells

**Figure 4:**
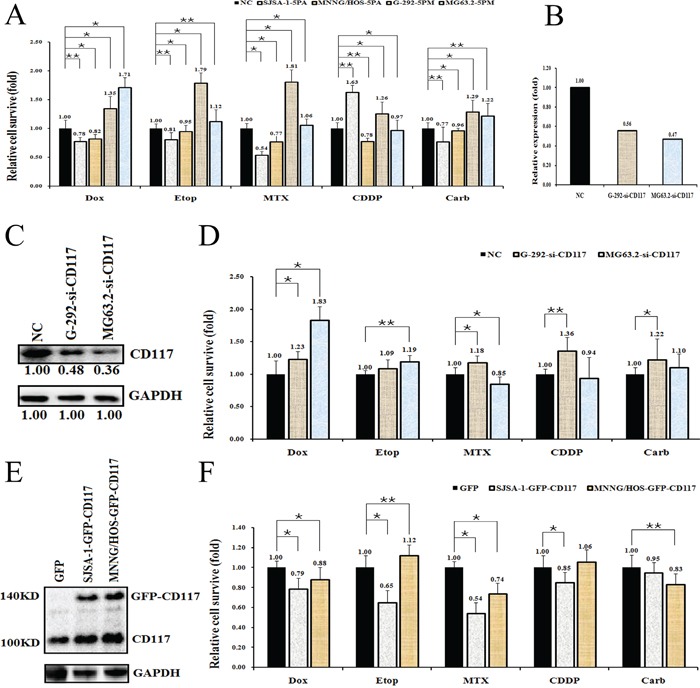
Effects of a forced reversal of the miR-34a-5p or CD117 levels on the drug resistance of G-292 and SJSA-1 cells Cell death triggered by an IC_50_ dose of drug in G-292, MG63.2, SJSA-1 and MNNG/HOS cells transfected with a miR-34a-5p mimic (5PM) or antagomiR (5PA) versus the negative control (NC) assayed 72 hr after treatment with the IC_50_ dose of drugs **A.** Levels of CD117 mRNA determined by qRT-PCR in the siRNA-transfected G-292 and MG63.2 cells versus the NC-transfected cells **B.** CD117 protein level (western blot analysis) in the siRNA-transfected versus the NC-transfected G-292 and MG63.2 cells **C.** Cell survival of the G-292 and MG63.2 cells transfected with siRNA relative to that of the NC-transfected G-292 and MG63.2 cells 72 hr after a treatment with the IC_50_ dose of drugs **D.** CD117 protein level (western blot analysis) in the GFP-tagged overexpression construct-transfected versus the NC-transfected SJSA-1 and MNNG/HOS cells **E.** Cell survival of the SJSA-1 and MNNG/HOS cells transfected with miR-34a-5p antagomiR (5PA) relative to that the NC-transfected SJSA-1 cells assayed 72 hr post-treatment with the IC_50_ dose of drugs **F.** *P value < 0.05; **P value < 0.01.

**Figure 5:**
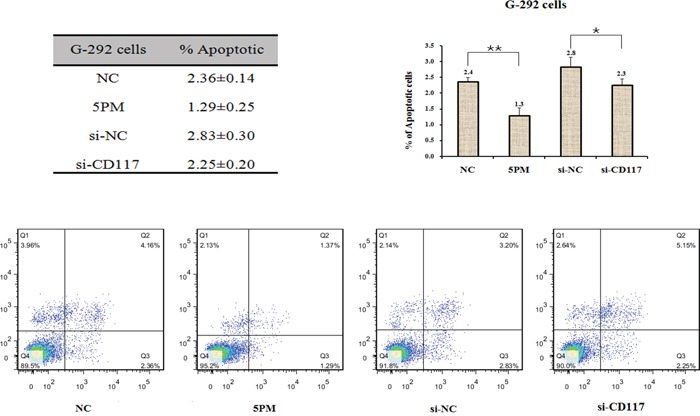
Effects of the forced reversal of both the miR-34a-5p and CD117 levels on the apoptosis of G-292 cells, with a graph of the analyzed data and plots of the original FACS data *P value < 0.05; **P value < 0.01.

The functional connection between miR-34a-5p and the CD117 gene in multi-drug resistance was then confirmed by comparing the effect on drug-triggered cell death in G-292 and MG63.2 cells transfected with the miR-34a-5p mimic and in SJSA-1 and MNNG/HOS cells transfected with the miR-34a-5p antagomiR. The transfection of the miR-34a-5p mimic into G-292 or MG63.2 cells significantly increased the drug resistance to all tested drugs, except MG63.2 to CDDP. By contrast, the transfection of the miR-34a-5p antagomiR into SJSA-1 or MNNG/HOS cells decreased the drug resistance to all tested drugs, except SJSA-1 to CDDP (Figure 4A). The si-CD117 was also transfected into G-292 or MG63.2 cells for testing the drug-resistance effect. The transfection of si-CD117 indeed decreased the level of CD117 at both the mRNA and protein level (Figure 4B and 4C). Following the changes in the CD117 level in G-292 and MG63.2 cells, the cell death triggered by all five drugs was reduced, except MG63.2 to MTX and CDDP (Figure 4D). Conversely, transfection of a GFP-tagged CD117 expression construct increased the CD117 protein level in SJSA-1 and MNNG/HOS cells (Figure 4E). The resulting drug resistance was significantly decreased for all five drugs, except MNNG/HOS to Etop and CDDP, whereas the transfection of only GFP showed a marginal effect (Figure 4F). In line with its negative effect on drug resistance (Figure 4D), the siRNA-mediated degradation of CD117 also reduced the number of apoptotic cells (Figure 5), this might suggest that the expression of CD117 reduces drug resistance by inhibiting cell proliferation *via* G2-phase arrest.

### MiR-34a-5p regulates the activities of the MEF2 signaling pathway in the context of OS multi-drug resistance

**Figure 6:**
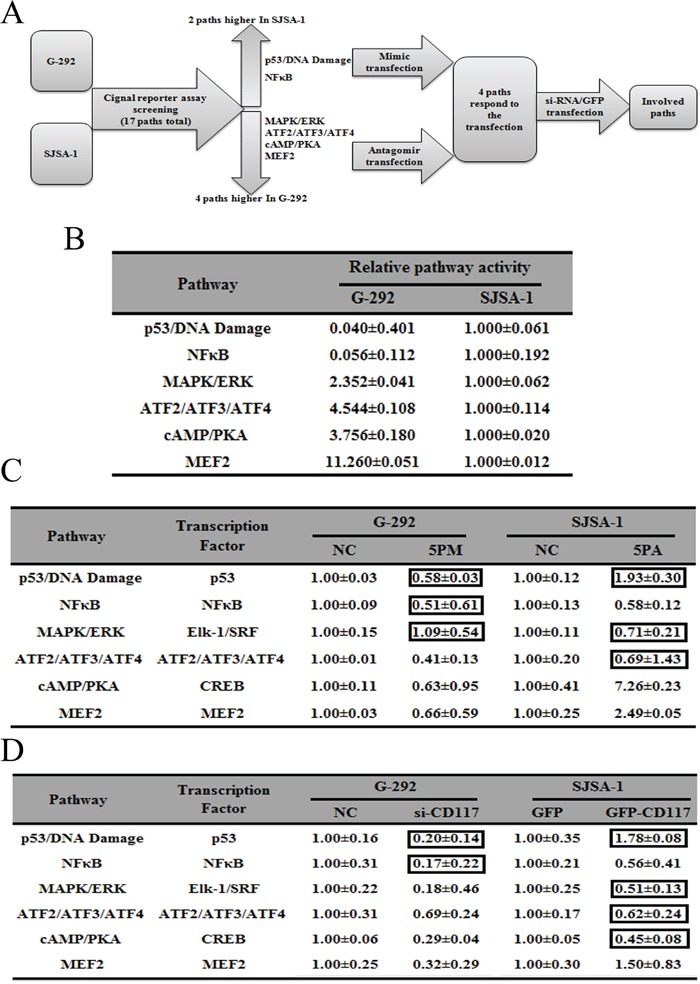
Signaling pathways regulated by miR-34a-3p and its downstream gene, CD117 Experimental scheme **A.** Relative activities (mean ± S.D.) of six pathways that differed by more than two-fold between G-292 and SJSA-1 cells **B.** Relative pathway activities in the miR-34a-5p mimic (5PM)-transfected cells versus the NC transfected G-292 cells and miR-34a-5p antagomiR (5PA)-transfected cells versus the NC-transfected SJSA-1 cells **C.** Relative pathway activities in the cells transfected with si-CD117 and GFP-CD117 versus the NC-transfected G-292 and SJSA-1 cells **D.** The boxes indicatethe pathways that failed to respond in an expected manner.

To further understand the underlying molecular mechanisms of OS drug resistance, we used both G-292 and SJSA-1 cells to measure the activities of the following six cancer-related signaling pathways: p53/DNA damage, NF-кB, MAPK/ERK, ATF2/ATF3/ATF4, cAMP/PKA and MEF2 (Figure 6A). The activities of all six pathways differed by more than two-fold between G-292 and SJSA-1 cells, which indicates that they might have a significant role in OS drug resistance. Among them, two pathways, p53/DNA damage and NF-кB, showed higher activities in SJSA-1 cells, whereas the other four pathways, MAPK/ERK, ATF2/ATF3/ATF4, cAMP/PKA and MEF2, showed higher activities in G-292 cells (Figure 6B). We then determined which of the six pathways were also affected by forced changes in the CD117 level in both G-292 and SJSA-1 cells. As shown in Figure 5, when the CD117 level was repressed by a miR-34a-5p mimic or si-CD117 in G-292 cells, the activities of ATF2/ATF3/ATF4, cAMP/PKA and MEF2 were all repressed, which correlates well with the positive regulation of these pathways by CD117 in G-292 cells (Figure 6C and 6D). We also transfected SJSA-1 cells with a miR-34a-5p antagomiR or the GFP-CD117 expression construct and measured the activities of these six pathways in SJSA-1 cells. Following the increase of the CD117 level, the NF-кB pathway was repressed whereas the MEF2 pathway was activated (Figure 6C and 6D), which is in accordance with the varying activities of these pathways between G-292 and SJSA-1 cells. Overall, only the MEF2 pathway was confirmed to be involved in the OS drug resistance mediated by the miR-34a-5p repression of the CD117 gene.

**Figure 7:**
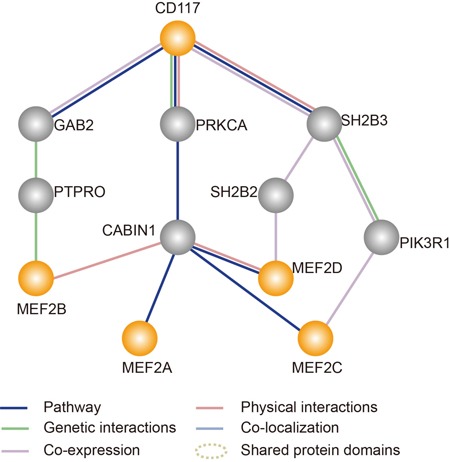
Simplified map showing an analysis of the interaction between the target gene (CD117) and the MEF2 pathway generated by GeneMANIA (http://genemania.org/) The orange solid lines specify a direct physical interaction among these proteins. The gray solid lines indicate genetic interactions among these proteins.

We searched for candidate interactions that could connect CD117 with the master transcription factor, MEF2 (Figure 7), using GeneMANIA [23]. In total, 18 interacting genes of CD117 were found in both GeneMANIA and the UniHI database [24]. Additional data mining was used to build a potential connection network describing the series of interactions (Figure 7). In this working model, the CD117 gene regulates the MEF2 signaling pathway *via* an interaction with three genes, GAB2, PRKCA and SH2B3.

### MiR-34a-5p promotes both the growth and Dox drug resistance of G-292 cells and SJSA-1-derived tumor xenografts in nude mice

**Figure 8:**
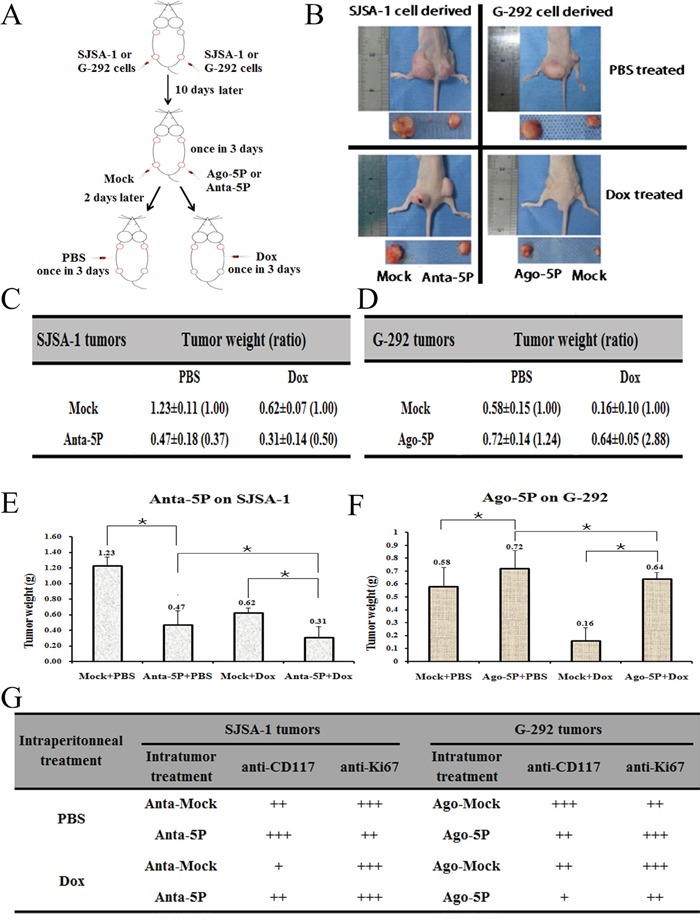
Effect of miR-34a-5p on the *in vivo* growth and Dox drug resistance of SJSA-1 and G-292-derived xenografts in nude mice **A.**, Experimental scheme: SJSA-1 or G-292 cells were subcutaneously injected at two points on the back of each nude mouse, with 2 sites/mouse, 6 mice for SJSA-1, and 6 mice for G-292. From the 10th day after cell injection, all six SJSA-1-generated tumors on the left back of the nude mice were intratumorally injected with 2 nM miR-34a-5p antagomiR, and the six right back sites were injected with 2 nM Mock; G-292-generated tumors on the left back of the nude mice were injected with 4Nm miR-34a-5p agomiR, and the six right sites were injected with 4 nM Mock; this process was repeated five times within 3 days. From the 25th day after cell injection, 3 SJSA-1 mice and 3 G-292 mice received Dox (2.5 mg/kg) intraperitoneally once every 3 days, for a total of 4 injections over 12 days. The remaining 6 mice (3 from SJSA-1 and 3 from G-292) received PBS as a mock treatment control. **B.**, Image of representative mice with tumors on day 40. **C., D., E and F.**, The mean ± SD of the tumor weight of the tumor for the same treatment was calculated, plotted (*, P value< 0.05), and summarized. **G.**, The protein levels of CD117 and Ki67 in each group were determined by immunostaining and summarized in the table (Magnification: 200×).

An intratumoral injection of miR-34a-5p agomiR/antagomiR, the scramble sequence control (Mock) or phosphate-buffered saline (PBS) into G-292/ SJSA-1-derived tumors was initiated on the tenth day and repeated five times over 3 days. The intraperitoneal injection of PBS or Dox began on day 25 in either the G-292 or SJSA-1 group and was repeated four times over 3 days (Figure 8A). The tumor mass was weighed at the end of this study. SJSA-1-derived tumors were approximately 2-fold heavier than G-292-derived tumors (Figure 8B–8F), suggesting that miR-34a-5p promotes tumor growth *in vivo*. Conversely, an intratumoral injection of the miR-34a-5p antagomiR into the SJSA-1 cell-derived tumor xenograft resulted in the opposite phenotype. A similar effect was observed for agomiR in G-292 tumors. Therefore, miR-34a-5p is capable of promoting *in vivo* tumor growth.

Consistent with the observation that SJSA-1 was more Dox resistant than G-292 cells in culture (Figure 1), after an intraperitoneal injection of Dox, the G-292 tumors were much smaller than the SJSA-1 tumors (Figure 8B). Furthermore, the tumor weight for the miR-34a-5p antagomiR/Dox in SJSA-1 mice was smaller than the weight for the miR-34a-5p antagomiR/Mock counterpart, and the opposite was true for the Dox-treated group of G-292 cells with or without miR-34a-5p agomiR transfection (Figure 8C–8F). These results indicated that miR-34a-5p compromised the tumor-inhibition capability of Dox.

Further confirmation of the miR-34a-5p role in the Dox resistance of OS came from the immunohistological analysis of the CD117 and Ki67 (an indicator of tumor cell proliferation) in the tumor sections of the Dox-treated versus PBS-treated mice (Figure 8G). The intratumoral injection of an miR-34a-5p agomiR (into G-292)/antagomiR (into SJSA-1) indeed led to the expected changes in the CD117 level in the tumor sections (Figure 8G), which confirmed that miR-34a-5p has a profound positive effect on both the growth and drug resistance of OS cell-derived tumor xenografts in nude mice.

## DISCUSSION

Our previous studies have shown that miR-193a-3p is involved in multi-drug resistance in both hepatocellular carcinoma [25] and bladder cancer [26, 27] *via* the repression of different target genes, including SRSF2, PLAU, HIC2 and LOXL4 [26, 27]. In the present work, we showed that miR-34a-5p is also involved in the multi-drug resistance of OS. MiR-34a-5p is up-regulated in multi-chemoresistant (SJSA-1 and MNNG/HOS) cell lines compared to the multi-chemosensitive (G-292 and MG63.2) OS cell lines (Figure 1 and 2). We next performed a comparative RNA-seq omic analysis of the SJSA-1, G-292 and MG63.2 cell lines and identified a panel of genes that are differentially expressed, including the CD117 gene, which exhibits a negative correlation with drug resistance (Figure 2). Both the roles and mechanisms of the CD117 gene in the context of OS drug resistance were systematically evaluated in both cultured cells and tumor xenografts in nude mice. Notably, miR-34a-5p was reported to suppress colorectal cancer metastasis and inhibit recurrence of colorectal cancer [28]. This suggests that miR-34a-5p might play distinct roles in different types of cancers. In addition, miR-34a was also found to inhibit the metastasis of osteosarcoma cells by repressing the expression of CD44, which indicates that miR-34a plays a tumor suppressor role [29]. Two different OS cells, the highly metastatic F5M2 and the low metastatic F4 cells were used to test the invasion and metastasis. In our work, we found that miR-34a-5p promotes the multi-drug resistance of OS by targeting the CD117 gene. Our results together with previous report suggest that miR-34a might play multi-functional roles by targeting different genes in the OS pathology. More investigations are needed to elucidate the fine regulation mechanism of miR-34a in OS pathology.

The CD117 gene encodes a type III RTK [19] and is an important mediator/initiator of several signaling cascades [30]. In addition, CD117 performs numerous functions in the tumorigenesis of several neoplasms, such as leukemia [31], glioblastoma [32], melanoma [33], and lung [34] and breast cancer [35]. Furthermore, a few studies have suggested that CD117 expression may be helpful in the differential diagnosis between high-grade neuroendocrine carcinomas of lungand non–small cell lung tumors [36, 37]. Despite the extensive studies of CD117, the role of CD117 in OS remains unclear. In this study, we clearly demonstrated that the down-regulation of CD117 mediated by miR-34a-5p might be one of the reasons for OS drug resistance. CD117 may also regulate other processes, including cell adhesion, differentiation and migration, which are significant for cancer development and treatment [38]. Here, we propose that CD117 mediates OS drug resistance *via* the MEF2 signaling pathway. The MEF2 pathway is activated in several types of cancer through different mechanisms. The detailed mechanisms for how CD117 affects the MEF2 pathway and the links between the MEF2 pathway and OS drug resistance still need to be elucidated. In addition, for the interaction of CD117 and miR-34a-5p with numerous signaling pathways, more detailed analyses are necessary to illuminate the detailed molecular mechanism of this regulation.

As previously reported, CD117 is a cancer stem cell marker and thus associated with increased drug resistance in several human cancers, including OS [39]. To investigate whether miR-34a-5p and CD117 affect the colony-forming ability of G-292 cells *in vitro*, we performed colony-forming and spheroids assays (supplementary Figure 1). Cells treated with miR-34a-5p formed clones and spheroids that were more numerous and larger than those of the control groups. In contrast, cells treated with si-CD117 formed much smaller clones and spheroids than did the control groups. As expected, the addition of the siRNAs for cancer stem markers, si-OCT4 or si-NANOG, decreased the size and quantity of the clones and spheroids. The results suggested that miR-34a-5p promotes cell proliferation, leading to the formation of larger clones, which is in accordance with the drug resistance-promoting effect. To test whether the promoting effect of miR-34a-5p-mediated cell proliferation is conducted by OCT4 and NANOG, we tested the expression levels of OCT4 and NANOG in OS cells transfected with either miR-34a-5p antagomiR or mimic. The transfection of miR-34a-5p antagomiR into SJSA-1 and MNNG/HOS cells significantly increased the expression of OCT4 and NANOG, whereas the transfection of miR-34a-5p mimic into G-292 or MG63.2 cells decreased the expression of OCT4 and NANOG (supplementary Figure 2). OCT4 and NANOG showed similar expression pattern with CD117 against miR-34a-5p antagomiR or mimic, which also indicates that the promoting effect of miR-34a-5p-mediated cell proliferation is a complicated event. The detailed mechanism remains to be clarified. Of note, CD117 showed a promoting effect on cell proliferation similar to that of the cancer stem cell markers OCT4 and NANOG, which also suggested that CD117 might function as a cancer stem marker in OS; however, CD117 showed a negative regulation of OS drug resistance in this study. CD117, as a potential cancer stem marker, could also impair OS drug resistance. More investigations are needed to elucidate the detailed mechanism for this complicated regulation in OS.

## MATERIALS AND METHODS

### Cell lines

The following seven OS cell lines were purchased from ATCC: G-292 (ATCC NO. CRL-1423), SJSA-1 (ATCC NO.CRL-2098), MG63 (ATCC NO.CRL-1427), Saos-2 (ATCC NO.HTB-85), U2OS (ATCC NO. 40342) and MNNG/HOS (ATCC NO. 1547). Another cell line, MG63.2, which was derived from MG63, was kindly provided by Dr. Luu from the University of Chicago [40]. All of the cell lines were cultured in Dulbecco's modified Eagle's medium (Invitrogen, Carlsbad, CA, USA) supplemented with 10% fetal bovine serum (Invitrogen) and 1% glutamine at 37°C in 5% CO_2_.

### RNA-seq analysis

RNA-seq analysis was performed by BGI-Tech of China, and RNA-seq library preparation and sequencing were performed by BGI (Shenzhen, China). Following purification, the RNA was fragmented using divalent cations at an elevated temperature, and first-strand cDNA was synthesized using random hexamer primers and Superscript TMIII (Invitrogen™, Carlsbad, CA, USA). Second-strand cDNA was synthesized using buffer, dNTPs, RNaseH, and DNA polymerase I. Short fragments were purified with a QiaQuick PCR extraction kit (Qiagen) and resolved with EB buffer for end repair and poly (A) addition. The short fragments were subsequently connected using sequencing adapters. After agarose gel electrophoresis, suitable fragments were used as templates for PCR amplification. During the QC steps, an Agilent 2100 Bioanalyzer and an ABI StepOnePlus Real-Time PCR System were used for the quantification and qualification of the sample library. Finally, the library (200 bp insert) was sequenced using an Illumina HiSeq 2000 (Illumina Inc., San Diego, CA, USA). The single-end library was prepared following the protocol of the Illumina TruSeq RNA Sample Preparation Kit (Illumina) [41].

### RNA analysis

Total RNA was isolated from the cells at the logarithmic phase using Trizol (Tiangen Biotech Co., Ltd., Beijing, China). For the mRNA analysis, cDNA primed by oligo-dT was made with a prime Script RT reagent kit (Tiangen Biotech Co., Ltd., Beijing, China), and the mRNA level of the CD117 gene was quantified using duplex-qRT-PCR analysis where the Taqman probes with different fluorescence for β-actin (provided by Shing Gene, Shanghai, China) were used in the FTC-3000P PCR instrument (Funglyn Biotech Inc., Canada). Using the 2^−ΔΔ^Ct method, normalization to the β-actin level was performed before the relative levels of the target genes were compared. The sequences of the primers and probes used for the qRT-PCR analysis are:

hCD117 F: 5′- AATATGAAGCATTCCCCAAACC-3′
hCD117 R: 5′- CTTCGGTGCCTTTTAATCTCG-3′
hCD117 probe: 5′-CY5- CACCAGCAGTGGATCTATATGAACAGAACC-3′
hACTB F: 5′-GCCCATCTACGAGGGGTATG-3′
hACTB R: 5′-GAGGTAGTCAGTCAGGTCCCG-3′
hACTB probe: 5′HEX-CCCCCATGCCATCCTG CGTC-3′

### Bulge-Loop™ miRNA qRT-PCR

For detecting and quantifying the expression of specific miRNAs, RNA was reverse transcribed using a Bulge-Loop™ miRNA qRT-PCR Primer Set (Ribobio) and quantified by SYBR Green-based real-time PCR analysis in the FTC-3000P (FUNGLYN BIOTECH INC., Canada). The Ct values of the target miRs were normalized to the Ct values of U6 RNA before quantification using the 2^−ΔΔ^Ct method.

### Chemotherapeutics

The chemotherapeutic drugs used in this paper are of clinical grade [26, 42, 43] (NCI Dictionary of Cancer Terms, http://www.cancer.gov/dictionary), Dox: doxorubicin (Haizheng, Zhejiang, China); Etop: etoposide (Hengrui, Jiangsu, China); MTX: Methotrexate (Lingnan, Guangdong, China); CDDP: cisplatin (Haosen, Jiangsu, China) and Carb: carboplatin (Qilu, Shandong, China).

### Drug resistance profiling (IC_50_ determination)

For a thiazolyl blue tetrazolium blue (MTT)-based cell proliferation assay, cells in the logarithmic phase of growth were seeded in triplicate in 96-well plates at a density of 0.5×10^4^ cells/well and treated with 4-fold serially diluted drugs for 72 hr. Then, 10 μl (5 mg/ml) of MTT salt (Sigma) was added to the corresponding well, the cells were incubated at 37°C for an additional 4 hr, and the reaction was stopped by lysing the cells with 150 μl of DMSO for 10 min. The optical density was measured at 570 nm. Both the linear regression parameters and the IC_50_ (the concentration of drug required to kill 50% of the cells) values with the no-drug control as the reference were calculated. The relative drug resistance was presented as the fold change in the IC_50_ of the cell lines relative to the lowest IC_50_.

### Transfection of the mimic/antagomiR/siRNA/overexpression plasmid

The mimic, agomiR, antagomiR, siRNA, scramble sequence control (NC) and riboFECT CP transfection kit were supplied by Guangzhou Ribobio, China. The expression construct for CD117 (EX-Z4357-M98) with a GFP tag was supplied by Guangzhou Fulengen, China. Transfection of both ribonucleic acid reagents or plasmids mentioned in this paper and the reporter plasmids in Cignal Finder™ Pathway Reporter Arrays (SABiosciences, USA) was performed according to the manufacturer's instructions. The sequences used in this study are as follows:

si-CD117:
5′-GGAUGAAACGAAUGAGAAU dTdT-3′
3′-dTdT CCUACUUUGCUUACUCUUA-5′
hsa-miR-34a-5p
antagomiR: 5′ACAACCAGCUAAGACACUGCCA 3′
mimics:
sense 5′UGGCAGUGUCUUAGCUGGUUGU 3′
antisense 5′ACAACCAGCUAAGACACUGCCA 3′

Chemically modified mimic oligonucleotides (agomiR) were synthesized to regulate the miR-34a-5p expression *in vivo*. The 3′ end of the oligonucleotides was conjugated to cholesterol, and all of the bases were 2′-OMe modified. The agomiR oligonucleotides were deprotected, desalted and purified by high-performance liquid chromatography.

### Luciferase reporter assay

A portion of the CD117 3′-UTR (552 bp, 760-1311 from a 2172-bp full length sequence) combined with the target sequence or the mutant target sequence for miR-34a-5p was cloned into the 3′ end of the luciferase-coding sequence of pGL3 (Invitrogen) to construct pGL3-luc-CD117 WT and pGL3-luc-CD117 Mut, respectively. The constructs were confirmed by DNA sequencing. Cells were seeded in 96-well plates at approximately 1×10^4^ cells per well and transfected with a mixture of 50 ng of pGL3-luc CD117 WT or Mut, 5 ng of Renilla plus 5 pmol of mimic or NC nucleotides, with the riboFECT CP transfection kit according to the manufacturer's instructions. Both the firefly and *Renilla* luciferase activities were measured 24 hr after transfection using the Dual-Luciferase Reporter Assay System (Promega) and a Promega GloMax 20/20 luminometer. The relative firefly luciferase activities of the UTR construct and pathway reporter constructs were analyzed as previously reported [26].

### Signaling pathway analysis

Constructs for the reporters of six signaling pathway, p53/DNA damage, NF-κB, MAPK/ERK, cAMP/PKA, ATF2/ATF3/ATF4 and MEF2 pathways, were obtained from SABiosciences (USA) and analyzed according to the manufacturer's instructions. Briefly, the cells were transfected in triplicate with each firefly luciferase reporter construct in combination with the Renilla luciferase-based control construct using the riboFECT CP transfection reagent, and both the luciferase activities were measured in the cell extracts 24 hr after transfection. The luciferase activities (luciferase unit) of the pathway reporter relative to those of the negative control in the transfected cells were calculated as a measurement of the pathway activity.

### Apoptosis analysis

Cells were harvested and rinsed with PBS twice. Then, 5 μl of FITC-labeled enhanced annexin V and 5 μl (20 μg/ml) of propidium iodide were added to a 100 μl cell suspension. After incubation in the dark for 15 min at room temperature, the samples were diluted with 100 μl of PBS. Flow cytometry was performed on a FACSCalibur instrument. The results were analyzed according to the manufacturer's instructions. The experiments were performed independently three times, and a representative is shown.

### Western blot analysis of protein

Cells were lysed with lysis buffer (60 mM Tris-HCl, pH 6.8, 2% SDS, 20% glycerol, 0.25% bromophenol blue, and 1.25% 2-mercaptoethanol) and heated at 100°C for 10 min before electrophoresis. Anti-CD117 (AM2137b) was purchased from Abgent, and anti-GAPDH (AM1020a) and HRP goat anti-mouse IgG antibody (LP1002a) were provided by Wuxiphama, Shanghai, China. The target bands were revealed by an enhanced chemiluminescence reaction (Pierce), and the density (level) of the proteins relative to the GAPDH band was quantified using a Gel-Pro Analyzer (Media Cybernetics).

### Bioinformatics analysis

CD117 and a representative gene, MEF2, in the MEF2 pathway were used as query genes to predict potential interactions using the GeneMANIA database (http://genemania.org/). GeneMANIA uses a fast heuristic algorithm to integrate functional association networks containing all input genes. To simplify the model, the shortest paths between query genes were extracted from the networks. We first chose the interactions reported in the literature to obtain the true functional role of each interaction in the connected network of all query genes.

### *In vivo* studies

Animal experiments were undertaken in accordance with the National Institutes of Health Guide for the Care and Use of Laboratory Animals. BALB/c male nude miceof 3-4 weeks of age were used for this study. SJSA-1 or G-292 cells were embedded in BD Matrigel™ Matrix (Becton, Dickinson, NJ, USA) and subcutaneously injected into the two sites on the backs of mice as follows: 4×10^6^ cells/site for SJSA-1, 1×10^7^ cells/site for G-292, 2 sites/mouse, 6 mice for SJSA-1, and 6 mice for G-292. From the 10th day after cell injection, all six SJSA-1-generated tumors on the left back of the nude mice were intratumorally injected with 2 nM miR-34a-5p antagomiR, and the six tumors on the right back were injected with 2 nM Mock. G-292-generated tumors on the left backs of nude mice were injected with 4 nM miR-34a-5p agomiR, and the six tumors on the right of the backs were injected with 4 nM Mock. These injections were repeated five times in 3 days. Three mice from SJSA-1 and 3 from G-292 intraperitoneally received Dox (2.5 mg/kg) once in 3 days. The remaining 6 mice (3 from SJSA-1 and 3 from G-292) received phosphate-buffered saline (PBS) as a mock treatment control, and these injections were repeated four times. The mice were humanely sacrificed on day 40, and the tumors were weighed and photographed. The tumor weight was reported as the mean ± S.D.

The expression of CD117 protein was measured using immunochemical analysis of 5-mm slices of formalin-fixed paraffin-embedded tumor xenografts from the nude mice. To avoid inter-treatment bias, the tissue slides from all six groups were placed on a single slide and simultaneously subjected to the same immunostaining. Antigens were retrieved by pretreating dewaxed sections in a microwave oven at 750 watts for 5 min in citrate buffer (pH 6) and processed with the Super Sensitive Link-Labeled Detection System (Biogenex, Menarini, Florence, Italy). The enzymatic activities were determined using 3-amino-9-ethylcarbazole (Dako, Milan, Italy) as a chromogenic substrate. Following counterstaining with Mayer hematoxylin (Invitrogen), the slides were mounted in aqueous mounting medium (glycergel, Dako). Pictures were taken usinga LEICA DM 4000B microscope, the relative level of each protein was calculated using LEICA software, and the percentage of Mock-treated relative to chemotherapeutic-treated tumors was calculated and plotted. The animal study proposal was approved by the Institutional Animal Care and Use Committee (IACUC) of the University of Science and Technology of China. All of the mouse experimental procedures were performed in accordance with the Regulations for the Administration of Affairs Concerning Experimental Animals approved by the State Council of People's Republic of China.

### Sphere formation assay

For induction of sphere formation, the G-292 cells were transfected with either a miR-34a-5p mimic or control oligo. after 24 hr, the cells were trypsinized, and 1×10^4^ cells were seeded in a six-well plate coated with attachment-preventing PolyHEMA (Sigma) using a 3 ml of sphere medium consisting of DMEM-F12 (Gibco) supplemented with B27 supplement (1:50, Gibco), EGF (20 ng/ml, Invitrogen), and bFGF (20 ng/ ml, Invitrogen), which was added to the medium every 3 days. Then, the sphere formation experiment was performed. The number of colonies larger than 50 μm in diameter was determined after ten days. Representative pictures were taken using a LEICA DM 4000B microscope at 40-fold magnification.

### Statistical analysis

The data are presented as the means, and the error bars indicate the S.D. All of the statistical analyses were performed using Excel (Microsoft, Redmond, WA, USA). A two-tailed Student's *t*-test, a one-way analysis of variance or Mann–Whitney *U* test was used to calculate statistical significance. A P value of <0.05 was considered significant.

## SUPPLEMENTARY FIGURES



## References

[1] Pillai RS, Bhattacharyya SN, Filipowicz W (2007). Repression of protein synthesis by miRNAs: how many mechanisms?. Trends in cell biology.

[2] Lu J, Getz G, Miska EA, Alvarez-Saavedra E, Lamb J, Peck D, Sweet-Cordero A, Ebert BL, Mak RH, Ferrando AA, Downing JR, Jacks T, Horvitz HR, Golub TR (2005). MicroRNA expression profiles classify human cancers. Nature.

[3] Volinia S, Calin GA, Liu CG, Ambs S, Cimmino A, Petrocca F, Visone R, Iorio M, Roldo C, Ferracin M, Prueitt RL, Yanaihara N, Lanza G, Scarpa A, Vecchione A, Negrini M A microRNA expression signature of human solid tumors defines cancer gene targets.

[4] Allen KE, Weiss GJ (2010). Resistance may not be futile: microRNA biomarkers for chemoresistance and potential therapeutics. Molecular cancer therapeutics.

[5] He H, Ni J, Huang J (2014). Molecular mechanisms of chemoresistance in osteosarcoma (Review). Oncology letters.

[6] Schetter AJ, Leung SY, Sohn JJ, Zanetti KA, Bowman ED, Yanaihara N, Yuen ST, Chan TL, Kwong DL, Au GK, Liu CG, Calin GA, Croce CM, Harris CC (2008). MicroRNA expression profiles associated with prognosis and therapeutic outcome in colon adenocarcinoma. Jama.

[7] Yang L, Li N, Wang H, Jia X, Wang X, Luo J (2012). Altered microRNA expression in cisplatin-resistant ovarian cancer cells and upregulation of miR-130a associated with MDR1/P-glycoprotein-mediated drug resistance. Oncology reports.

[8] Song B, Wang Y, Xi Y, Kudo K, Bruheim S, Botchkina GI, Gavin E, Wan Y, Formentini A, Kornmann M, Fodstad O, Ju J (2009). Mechanism of chemoresistance mediated by miR-140 in human osteosarcoma and colon cancer cells. Oncogene.

[9] Nakatani F, Ferracin M, Manara MC, Ventura S, Del Monaco V, Ferrari S, Alberghini M, Grilli A, Knuutila S, Schaefer KL, Mattia G, Negrini M, Picci P, Serra M, Scotlandi K (2012). miR-34a predicts survival of Ewing's sarcoma patients and directly influences cell chemo-sensitivity and malignancy. The Journal of pathology.

[10] Gao J, Li N, Dong Y, Li S, Xu L, Li X, Li Y, Li Z, Ng SS, Sung JJ, Shen L, Yu J (2015). miR-34a-5p suppresses colorectal cancer metastasis and predicts recurrence in patients with stage II/III colorectal cancer. Oncogene.

[11] Hermeking H (2007). p53 enters the microRNA world. Cancer cell.

[12] Yamakuchi M, Lowenstein CJ (2009). MiR-34, SIRT1 and p53: the feedback loop. Cell Cycle.

[13] Wu J, Wu G, Lv L, Ren YF, Zhang XJ, Xue YF, Li G, Lu X, Sun Z, Tang KF (2012). MicroRNA-34a inhibits migration and invasion of colon cancer cells via targeting to Fra-1. Carcinogenesis.

[14] Ji Q, Hao X, Meng Y, Zhang M, Desano J, Fan D, Xu L (2008). Restoration of tumor suppressor miR-34 inhibits human p53-mutant gastric cancer tumorspheres. BMC cancer.

[15] Li N, Fu H, Tie Y, Hu Z, Kong W, Wu Y, Zheng X (2009). miR-34a inhibits migration and invasion by down-regulation of c-Met expression in human hepatocellular carcinoma cells. Cancer letters.

[16] Gallardo E, Navarro A, Vinolas N, Marrades RM, Diaz T, Gel B, Quera A, Bandres E, Garcia-Foncillas J, Ramirez J, Monzo M (2009). miR-34a as a prognostic marker of relapse in surgically resected non-small-cell lung cancer. Carcinogenesis.

[17] Botter SM, Neri D, Fuchs B (2014). Recent advances in osteosarcoma. Current opinion in pharmacology.

[18] Yang J, Zhang W (2013). New molecular insights into osteosarcoma targeted therapy. Current opinion in oncology.

[19] Ni S, Huang D, Chen X, Huang J, Kong Y, Xu Y, Du X, Sheng W (2012). c-kit gene mutation and CD117 expression in human anorectal melanomas. Human pathology.

[20] Went PT, Dirnhofer S, Bundi M, Mirlacher M, Schraml P, Mangialaio S, Dimitrijevic S, Kononen J, Lugli A, Simon R, Sauter G (2004). Prevalence of KIT expression in human tumors. Journal of clinical oncology.

[21] Pon JR, Marra MA (2016). MEF2 transcription factors: developmental regulators and emerging cancer genes. Oncotarget.

[22] Siemens H, Jackstadt R, Kaller M, Hermeking H (2013). Repression of c-Kit by p53 is mediated by miR-34 and is associated with reduced chemoresistance, migration and stemness. Oncotarget.

[23] Montojo J, Zuberi K, Rodriguez H, Bader GD, Morris Q (2014). GeneMANIA: Fast gene network construction and function prediction for Cytoscape. F1000Research.

[24] Kalathur RK, Pinto JP, Hernandez-Prieto MA, Machado RS, Almeida D, Chaurasia G, Futschik ME (2014). UniHI 7: an enhanced database for retrieval and interactive analysis of human molecular interaction networks. Nucleic acids research.

[25] Ma K, He Y, Zhang H, Fei Q, Niu D, Wang D, Ding X, Xu H, Chen X, Zhu J (2012). DNA methylation-regulated miR-193a-3p dictates resistance of hepatocellular carcinoma to 5-fluorouracil via repression of SRSF2 expression. The Journal of biological chemistry.

[26] Lv L, Deng H, Li Y, Zhang C, Liu X, Liu Q, Zhang D, Wang L, Pu Y, Zhang H, He Y, Wang Y, Yu Y, Yu T, Zhu J (2014). The DNA methylation-regulated miR-193a-3p dictates the multi-chemoresistance of bladder cancer via repression of SRSF2/PLAU/HIC2 expression. Cell death & disease.

[27] Deng H, Lv L, Li Y, Zhang C, Meng F, Pu Y, Xiao J, Qian L, Zhao W, Liu Q, Zhang D, Wang Y, Zhang H, He Y, Zhu J (2014). miR-193a-3p regulates the multi-drug resistance of bladder cancer by targeting the LOXL4 gene and the Oxidative Stress pathway. Molecular cancer.

[28] Gao J, Li N, Dong Y, Li S, Xu L, Li X, Li Y, Li Z, Ng SS, Sung JJ, Shen L, Yu J (2015). miR-34a-5p suppresses colorectal cancer metastasis and predicts recurrence in patients with stage II/III colorectal cancer. Oncogene.

[29] Zhao H, Ma B, Wang Y, Han T, Zheng L, Sun C, Liu T, Zhang Y, Qiu X, Fan Q (2013). miR-34a inhibits the metastasis of osteosarcoma cells by repressing the expression of CD44. Oncology reports.

[30] Anderson DM, Lyman SD, Baird A, Wignall JM, Eisenman J, Rauch C, March CJ, Boswell HS, Gimpel SD, Cosman D (1990). Molecular cloning of mast cell growth factor, a hematopoietin that is active in both membrane bound and soluble forms. Cell.

[31] Ikeda H, Kanakura Y, Tamaki T, Kuriu A, Kitayama H, Ishikawa J, Kanayama Y, Yonezawa T, Tarui S, Griffin JD (1991). Expression and functional role of the proto-oncogene c-kit in acute myeloblastic leukemia cells. Blood.

[32] Mahzouni P, Jafari M (2012). The study of CD117 expression in glial tumors and its relationship with the tumor-type and grade. J Res Med Sci.

[33] Montone KT, van Belle P, Elenitsas R, Elder DE (1997). Proto-oncogene c-kit expression in malignant melanoma: protein loss with tumor progression. Mod Pathol.

[34] Krystal GW, Hines SJ, Organ CP (1996). Autocrine growth of small cell lung cancer mediated by coexpression of c-kit and stem cell factor. Cancer research.

[35] Hines SJ, Organ C, Kornstein MJ, Krystal GW (1995). Coexpression of the c-kit and stem cell factor genes in breast carcinomas. Cell growth & differentiation.

[36] Arber DA, Tamayo R, Weiss LM (1998). Paraffin section detection of the c-kit gene product (CD117) in human tissues: value in the diagnosis of mast cell disorders. Human pathology.

[37] Gibson PC, Cooper K (2002). CD117 (KIT): a diverse protein with selective applications in surgical pathology. Advances in anatomic pathology.

[38] Sattler M, Salgia R (2004). Targeting c-Kit mutations: basic science to novel therapies. Leukemia research.

[39] Adhikari AS, Agarwal N, Wood BM, Porretta C, Ruiz B, Pochampally RR, Iwakuma T (2010). CD117 and Stro-1 identify osteosarcoma tumor-initiating cells associated with metastasis and drug resistance. Cancer research.

[40] Su Y, Luo X, He BC, Wang Y, Chen L, Zuo GW, Liu B, Bi Y, Huang J, Zhu GH, He Y, Kang Q, Luo J, Shen J, Chen J, Jin X (2009). Establishment and characterization of a new highly metastatic human osteosarcoma cell line. Clinical & experimental metastasis.

[41] Tarazona S, Garcia-Alcalde F, Dopazo J, Ferrer A, Conesa A (2011). Differential expression in RNA-seq: a matter of depth. Genome research.

[42] Heiser LM, Sadanandam A, Kuo WL, Benz SC, Goldstein TC, Ng S, Gibb WJ, Wang NJ, Ziyad S, Tong F, Bayani N, Hu Z, Billig JI, Dueregger A, Lewis S, Jakkula L Subtype and pathway specific responses to anticancer compounds in breast cancer.

[43] Andrisano V, Bartolini M, Gotti R, Cavrini V, Felix G (2001). Determination of inhibitors' potency (IC50) by a direct high-performance liquid chromatographic method on an immobilised acetylcholinesterase column. Journal of chromatography B, Biomedical sciences and applications.

