# Correction: MiR-34a-5p promotes the multi-drug resistance of osteosarcoma by targeting the CD117 gene

**DOI:** 10.18632/oncotarget.20599

**Published:** 2017-09-01

**Authors:** Shanbao Cai, Youguang Pu, Fangfang Zhao, Haiyan Wang, Wenjing Cai, Jin Gao, Yinpeng Li

**Affiliations:** ^1^ Cancer Epigenetics Program, Anhui Cancer Hospital, West District of Anhui Provincial Hospital, Hefei 230031, Anhui, China; ^2^ Department of Clinical Geriatrics, Anhui Provincial Hospital, Hefei 230031, Anhui, China; ^3^ Indiana University School of Medicine, Indianapolis, IN 46202, USA; ^4^ Department of Radiation Oncology, Anhui Cancer Hospital, West District of Anhui Provincial Hospital, Hefei 230031, Anhui, China; ^5^ Xinxiang Medical University, Xinxiang 453000, Henan, China; ^6^ Department of Orthopedic Surgery, Anhui Cancer Hospital, West District of Anhui Provincial Hospital, Hefei 230031, Anhui, China

**This article has been corrected:** Dr. Shanbao Cai was moved to the first position in the author list.

The authors sincerely apologize for this oversight.

Original article: Oncotarget. 2016; 7:28420-28434. https://doi.org/10.18632/oncotarget.8546

