# Supplementary material for: MiR-34a-5p promotes the multi-drug resistance of osteosarcoma by targeting the CD117 gene
Source: Oncotarget. 2016 Apr 1;7(19):28420–34. doi: 10.18632/oncotarget.8546 (PMC5053736; doi:10.18632/oncotarget.8546)
Supplement: Supplementary file 1 [file oncotarget-07-28420-s001.pdf]

# MiR-34a-5p promotes the multi-drug resistance of osteosarcoma by targeting the CD117 gene

## SUPPLEMENTARY FIGURES

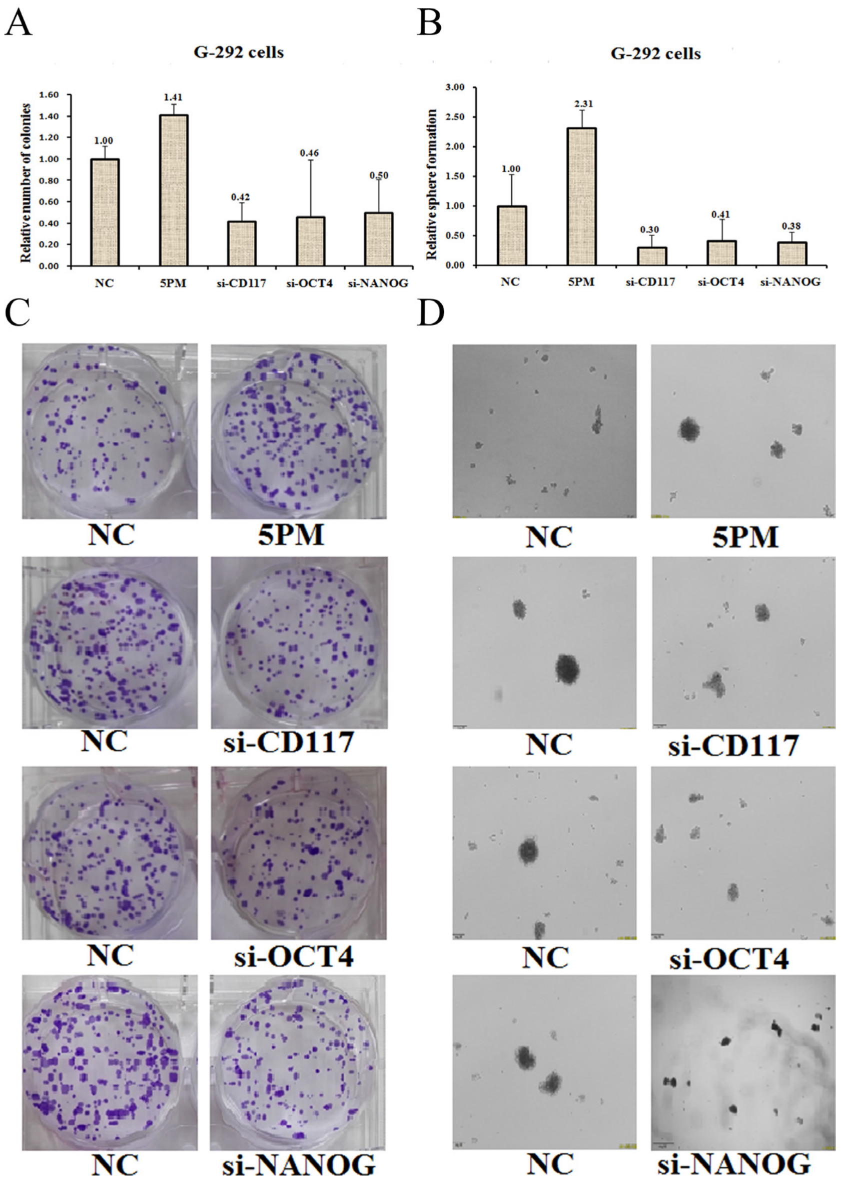


**Supplementary Figure S1: Role of the miR-34a-5p/CD117 axis in colony formation and spheroids in G-292 cells.** G-292 cells were treated for 24 hr with NC, 5PM, si-CD117, si-OCT4, and si-NANOG and then treated with SCF (or water) and subjected to a sphere formation assay. The sphere numbers were determined after seven days for the first generation (G1) and seven days after seeding for G2. Treatment with SCF was repeated when the cells were passaged. Colony formation numbers, relative sphere formation **A** and **C.**, respectively) and representative pictures **B** and **D.** are shown. The results represent the mean ± SD (n=3) (Magnification: 40×).

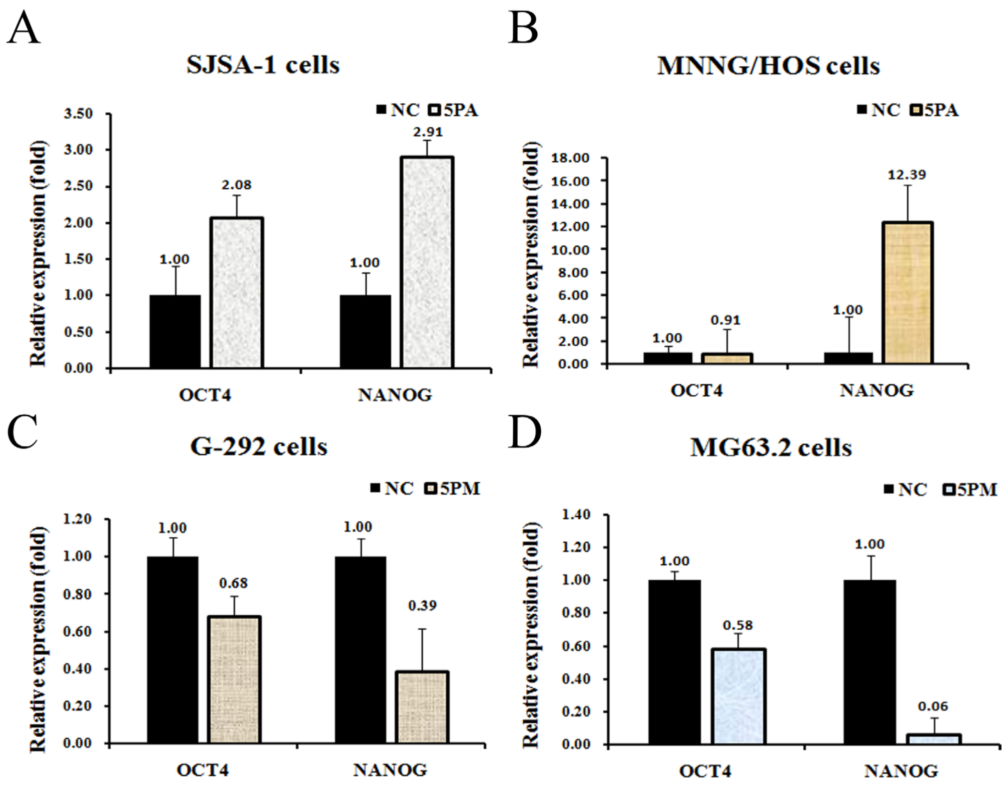


**Supplementary Figure S2: The OCT4 and NANOG levels in the miR-34a-5p mimic (5PM)-transfected G-292 and MG63.2 cells, and in the miR-34a-5p antagomiR (5PA)-transfected SJSA-1 and MNNG/HOS cells versus the negative control (NC) cells determined by qRT-PCR.**
